# How to choose the best control strategy? Mathematical models as a tool for pre-intervention evaluation on a macroparasitic disease

**DOI:** 10.1371/journal.pntd.0008789

**Published:** 2020-10-22

**Authors:** Elisa Fesce, Claudia Romeo, Eleonora Chinchio, Nicola Ferrari

**Affiliations:** 1 Department of Veterinary Medicine, Università degli Studi di Milano, Milan, Italy; 2 Centro di Ricerca Coordinata Epidemiologia e Sorveglianza Molecolare delle Infezioni, Università degli Studi di Milano, Milan, Italy; Chinese Center for Disease Control and Prevention, CHINA

## Abstract

During the last century, emerging diseases have increased in number, posing a severe threat for human health. Zoonoses, in particular, represent the 60% of emerging diseases, and are a big challenge for public health due to the complexity of their dynamics. Mathematical models, by allowing an *a priori* analysis of dynamic systems and the simulation of different scenarios at once, may represent an efficient tool for the determination of factors and phenomena involved in zoonotic infection cycles, but are often underexploited in public health. In this context, we developed a deterministic mathematical model to compare the efficacy of different intervention strategies aimed at reducing environmental contamination by macroparasites, using raccoons (*Procyon lotor*) and their zoonotic parasite *Bayilsascaris procyonis* as a model system. The three intervention strategies simulated are raccoon depopulation, anthelmintic treatment of raccoons and faeces removal. Our results show that all these strategies are able to eliminate the parasite egg population from the environment, but they are effective only above specific threshold coverages. Host removal and anthelmintic treatment showed the fastest results in eliminating the egg population, but anthelmintic treatment requires a higher effort to reach an effective result compared to host removal. Our simulations show that mathematical models can help to shed light on the dynamics of communicable infectious diseases, and give specific guidelines to contain *B*. *procyonis* environmental contamination in native, as well as in new, areas of parasite emergence. In particular, the present study highlights that identifying in advance the appropriate treatment coverage is fundamental to achieve the desired results, allowing for the implementation of cost- and time-effective intervention strategies.

## Author summary

Emerging infectious diseases involve complex systems on which background information is often lacking. Mathematical modelling, by providing a dynamic analysis of interactions between populations, can help our understanding of a disease transmission and maintenance, allowing for the simulation and comparison of intervention strategies aimed at controlling disease spread. We applied mathematical modelling to *baylisascariasis*, a disease caused by accidental ingestion of *Baylisascaris procyonis* eggs by humans, with consequences including fatal or severe neurologic disease. *B*. *procyonis* is an intestinal roundworm of raccoons, which shed eggs in the environment with their faeces. We modelled this system to simulate three different ways to reduce egg contamination and exposure risk for humans: raccoon depopulation, anthelmintic treatment of raccoons and faeces removal. Our models revealed that raccoon depopulation is the most efficient strategy to eliminate *B*. *procyonis* eggs, providing specific information on the minimum effort required to achieve their elimination, and showing that mathematical modelling is an efficient tool to identify the best strategy to control a disease beforehand, without wasting time and resources.

## Introduction

During the last century, the total number of infectious diseases has decreased globally, but an opposite trend has been observed for emerging and re-emerging diseases, which have increased in number and currently threaten human health [1–3]. Several diseases, like MERS, SARS, chikungunya and Ebola, emerged in recent years, causing severe epidemics and requiring a coordinated global response in terms of continued surveillance and research [1,4–6]. More recently, the COVID-19 pandemic dramatically and urgently highlighted the threat posed by emerging diseases to human health [7].

The development of efficient and tailored intervention strategies to control such diseases is thus essential, but the lack of previous field data, of background information on neglected or unknown pathogens, and the complexity of some systems, make the choice of the most efficient intervention strategy challenging. Additionally, most emerging infectious diseases (EIDs) are zoonoses (the 60.3% of EIDs), and most of them (71.8%) originate in wildlife [2]. Monitoring infected wild animals and identifying their role in the spread and maintenance of diseases complicates things further [8,9]. The range of approaches to cope with wildlife-originated zoonoses is wide, and most of applied strategies and techniques still give controversial results, often leading to a waste of time and resources [8–11]. As a consequence, on the one hand it is necessary to improve the current knowledge on emerging infectious diseases and their dynamics, on the other hand it is fundamental to develop tools for a better evaluation of the efficacy of potential intervention strategies.

Mathematical models can represent an efficient tool for an *a priori* simulation of host-pathogen interactions, thus improving our understanding of infectious diseases dynamics and helping to assess and evaluate different approaches to control diseases [12–15]. Mathematical models have been widely used in the investigation of disease dynamics and basic reproductive ratio (R_0_) role in disease spread and maintenance. They have been used also to understand the role of different sources of heterogeneities in host populations in affecting the transmission and maintenance of diseases [12–14,16–19]. However, despite their potential, the use of mathematical models in empirical epidemiological studies and in the planning of public health policies still has limited practical application [20].

For this reason, we used a macroparasitic zoonotic disease as a model to apply and adapt a consolidated mathematical model [12] in order to provide a framework for the analysis of efficacy and efficiency of different intervention strategies.

*B*. *procyonis* is an ascarid nematode that infects North American raccoons as natural definitive hosts. The infective stage of the parasite is represented by eggs shed in the environment with raccoons' faeces, where they become infectious and can remain viable for years [21,22]. Birds and other mammals, humans included, may accidentally become infected and act as paratenic hosts [20,23–26]. Like with most of ascarids, ingestion of eggs by paratenic hosts may result in *larva migrans* syndrome, with larvae migrating from the gut and encysting into various host tissues. Compared to other ascarids, *larva migrans* by *B*. *procyonis* is particularly aggressive, often causing extensive neural damage. Several cases of severe or fatal neural *larva migrans* syndrome have been reported in humans in the last decades, most of them in children [21,25,27], and *baylisascariasis* is now considered an emerging zoonosis [23]. The opportunistic behaviour of raccoons, that often coexist with humans in urban, suburban, and rural environments, combined with the high number of eggs shed and with their resistance, leads to extensive opportunities for contact and infection of human beings [21,22,28].

The sanitary relevance of *B*. *procyonis* and the high exposure risk make the development of prevention strategies for *B*. *procyonis* infection in humans necessary. Three main approaches are currently taken into consideration: the active removal of raccoons [21,29], the treatment of raccoons with anthelmintic baits [21,30,31], and the reduction of environmental contamination through faeces removal [21,29]. Each one of these strategies presents some strengths and weaknesses [21,26,29,30], but a systematic comparison of the efficacy (i.e. the capacity of the treatment to reach the egg elimination from the environment), and efficiency (i.e. the time needed to reach the egg elimination) of the strategies is still lacking. For this reason, we propose the use of a pre-existent and already validated mathematical model to (i) investigate the dynamics of the raccoon-B.*procyonis* system, and, in particular, (ii) compare both the efficacy and the efficiency of the three above-mentioned intervention strategies.

## Methods

### Study system

The investigated system consists of three interacting populations: the host population (raccoons, H), the parasite population (adult *B*. *procyonis*, P) and the free-living infective stage of the parasite (*B*. *procyonis* eggs, E). The simulated intervention strategies are: (i) raccoon depopulation, (ii) anthelmintic treatment and (iii) faeces removal.

Firstly, we performed a preliminary sensitivity analysis of the parameters representing the different intervention strategies, to compare their influence on the number of environmental eggs [32]. Then, we analysed the system by combining the analysis of equilibria (S1 Text) and simulations to evaluate both the efficacy and the efficiency of each intervention strategy. Multiple simulations were performed using the simple Euler forward integration method (function *euler* of”deSolve” package in R 3.6.3 software). In order to assess efficacy, we evaluated whether each specific intervention strategy was able to eliminate the egg population within 50 years. Efficacy was computed by using the equations for the analysis of the system equilibria reported in S1 Text. To assess the efficiency of each intervention strategy we evaluated the effective time needed to reach the new steady state. Since we focused our attention on intervention strategies eliminating the egg population and both adult parasite and egg populations consist of a discrete number of individuals, we considered that an intervention strategy reaches the steady state when the computed number of eggs and parasites is lower than one.

We chose to consider a 50-years time frame to clearly show and compare the effects of the intervention strategies on both the number of eggs and the time needed to reach the equilibrium. For this reason, interventions requiring more than 50 years to reach the equilibrium were not considered in the analysis and we only reported the number of eggs reached at the 50^th^ year.

To simplify the comparison between intervention strategies, we will focus hereafter on the proportion of subjects (raccoons/parasites/eggs, depending on the intervention strategy) treated per day, expressed as a percentage of the whole population size on that day and named hereafter “treatment coverage”. It must be noted that the proportion of subjects treated does not strictly represent a constant number of raccoons/parasites/eggs treated per day, because it will depend on the population size of that day.

Due to the recent introduction of raccoons and *B*. *procyonis* in areas outside their natural North and Central American distribution range [21], two different scenarios have been explored:

- The ‘native population’ scenario: represented by a raccoon population in its native range, where the host population is close to its environmental carrying capacity (K) and the system is close to its steady state.
- The ‘introduced population’ scenario: represented by a raccoon population recently introduced in a new area, where neither the environmental carrying capacity nor the system’s steady state have been reached yet. In this scenario, we considered, as initial sizes of H, P and L, the values reached from the system when the host population reaches 50% of its environmental carrying capacity.

We considered these two scenarios to take into account their epidemiological and demographic differences, as they differently affect the feasibility and efficacy of management and intervention strategies.

### Dynamics of the system: mathematical model

Following Anderson and May [12], two deterministic models based on a system of three coupled differential equations have been implemented. Firstly, with the base model, we explored the dynamics of the system without any intervention, and secondly we introduced into the system the intervention strategies aimed at eliminating environmental *B*. *procyonis* eggs.

**Fig 1:**
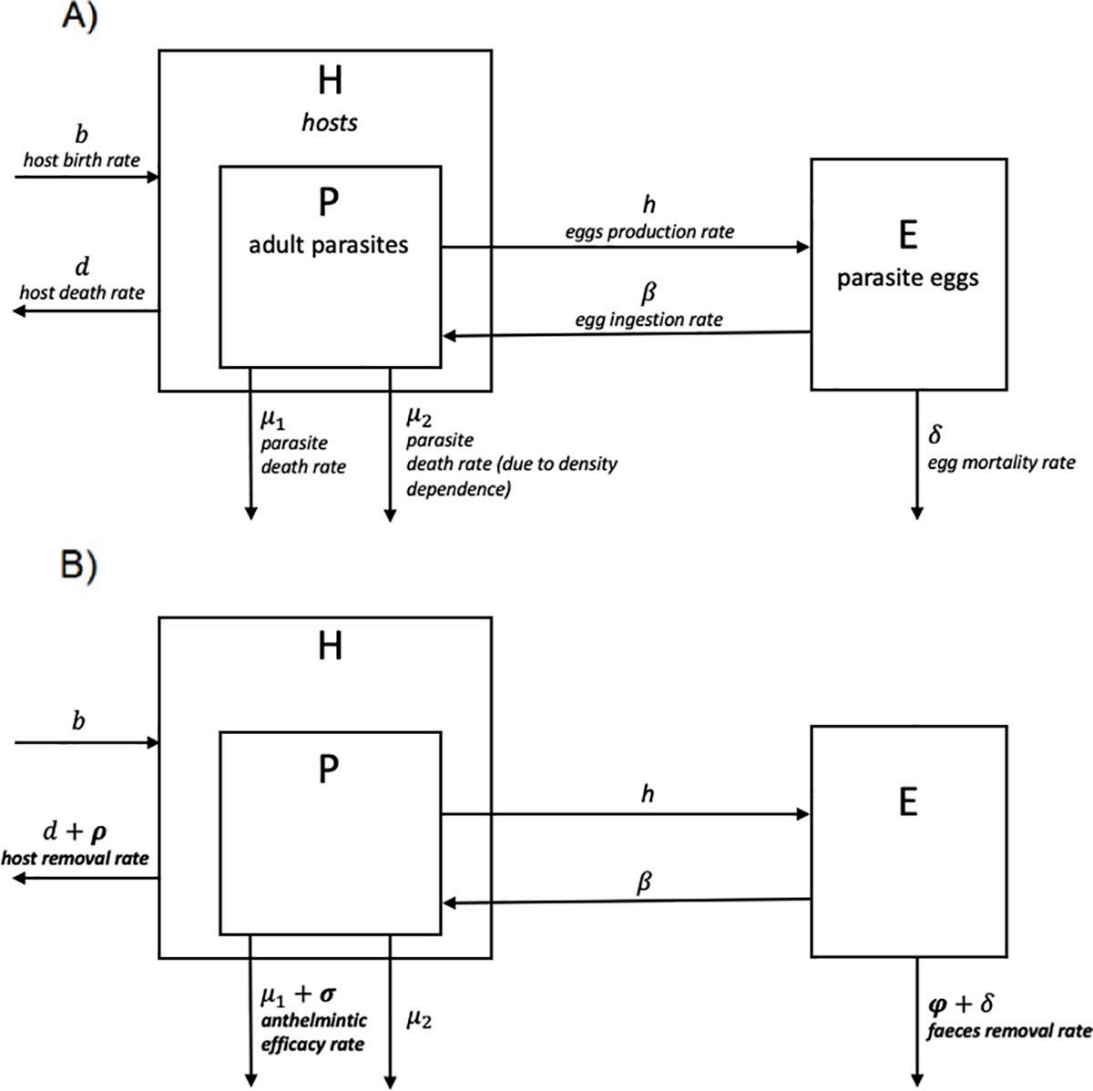
Modelled interactions between raccoons (*Procyon lotor*) and *Baylisascaris procyonis*. (A) Schematic representation of the interaction between *P*. *lotor*, the adult stage of the parasite *B*. *procyonis* and its free-living stages, illustrating the biological processes included in the model. (B) Schematic representation of the interaction between *P*. *lotor* and *B*. *procyonis*, their biological processes, biological parameters included in the model and the simulated intervention strategies.

The dynamics of the base model, without any human intervention, can be described according to the following system of equations and are represented by the flow chart in Fig 1A:

$$\left\{ \begin{array}{l} \frac{dH}{dt}=(b-d)H(\frac{K-H}{K})\\ \frac{dP}{dt}=\beta EH-(d+\mu_{1})P-\mu_{2}H(\frac{P^{2}}{H^{2}}\frac{k+1}{k}+\frac{P}{H})\\ \frac{dE}{dt}=hP-\beta EH-\delta E \end{array}\right.$$ (1)

In this model, host population size (Eq 1) increases with raccoons’ birth rate (*b*) and decreases with death rate (*d*). Density-dependence in host population growth is taken into account by including in the model a fixed carrying capacity of the environment (*K*). Since *B*. *procyonis* impacts on raccoon health are rarely described and age resistance and/or intestinal immunity with self-cure are considered to be the main limiting process on *B*. *procyonis* number in raccoons [21], we assumed that the effect of the parasite on host survival and reproduction at the population level is negligible, and thus host population size is not affected by the parasite. Parasite population (Eq 1) increases through the rate of ingestion (*β*) of infective eggs by the hosts, and decreases due to the combined effects of parasite death rate (*μ*_*1*_), host death rate (*d*), and parasite density-dependent mortality (*μ*_*2*_), which depends in turn on the aggregated distribution of parasites within the host population [33]. The parameter *k* affecting parasite density-dependent mortality provides an inverse measure of the extent of parasite aggregation [34]. Under natural conditions, raccoons may acquire infections even through predation of paratenic infected host, such as small mammals, but because of the central role of raccoon latrines in the transmission dynamics of *B*. *procyonis* [35], here we considered infection only through ingestion of environmental eggs. Finally, egg population size (Eq 1) increases with adult female parasites fecundity rate (h), and decreases through both natural egg mortality (*δ*), and host ingestion rate (*β*). Because of the lack of information on the effective proportion of ingested infective larvae that develop to the adult stages within the host, we considered all ingestions of eggs resulting in a successful parasite establishment.

### Simulation of intervention strategies

In order to explore the effects of intervention strategies on *B*. *procyonis* eggs, the base model introduced above has been modified as described by the following system of equations and by Fig 1B:

$$\left\{ \begin{array}{l} \frac{dH}{dt}=(b-d)H(\frac{K-H}{K})-\rho H\\ \frac{dP}{dt}=\beta EH-(d+\mu_{1}+\sigma +\rho )P-\mu_{2}H(\frac{P^{2}}{H^{2}}\frac{k+1}{k}+\frac{P}{H})\\ \frac{dL}{dt}=hP-\beta EH-(\delta +\varphi )E \end{array}\right.$$ (2)

Raccoon depopulation affects the system by removing hosts and the parasites they harbour by a quantity that depends on the host removal rate (*ρ*). Anthelmintic treatment, by killing adult parasites harboured in raccoons by a quantity that depends on the anthelmintic administration rate (*σ*), only affects the parasite population. Similarly faeces removal, only acts on the egg population by decreasing its size by a quantity that depends on faeces removal rate (*φ)*.

It is important to notice that once host population becomes extinct, the parasite population will die out too, reducing the model to the following equation:

$$\frac{dE}{dt}=-\delta E$$ (3)

### Parameters estimation

Parameters used in the simulations were derived from published data. The mean lifespan of *P*. *lotor* is assumed as 2.3 years (839.5 days) [36], and the mean number of offspring as 3.8 youngs/female per year (0.01041/day) [35]. Thus, considering a sex ratio of 1:1 [37], daily host birth rate *b* is 0.0052, and daily host mortality *d* is 0.00123/day. Population density of racoons is highly dependent on habitat conditions, with reported values ranging from 1 to 100 raccoons/km^2^ in the wild and exceeding 100/km^2^ in urban areas [38–40]. For this reason, for both scenarios we arbitrarily chose an environmental carrying capacity (K) of 1000, in order to simulate a plausible population unit of intervention for both an introduced and a native raccoon population. We estimated *B*. *procyonis* lifespan based on data available for closely related ascarid nematodes such as *Ascaris lumbriocoides* and *Baylisascaris shroederi*. Both these species have a 1–2 years lifespan (365–730 days) [41,42], thus daily death rate of adult parasites *μ*_*1*_ was set at 0.001369 individuals/day. We used 0.393 as parameter of parasite aggregation (*k*), in accordance with the measure estimated for the ascarid *Toxocara cati* [43]. The reproductive output of a single *B*. *procyonis* female in one day (*h*) is reported to be 179,000 eggs/day [21,44] and incorporates egg production rate, scaled by the development time from egg to infecting stage. Egg mortality rate δ was chosen in order to allow a complete extinction of eggs within 5 years [45] when simulating with Eq (3) and an initial egg number of 7.97x10^7^ eggs (i.e. the number of eggs at the equilibrium of the system). Since the estimation of μ_2_ and β from literature data was unfeasible, we arbitrarily identified those values with successive simulations, aiming at the achievement of the equilibria of the system with a mean abundance for *B*. *procyonis* of 15 parasites/host [21].

### Intervention strategy rates

Host removal rate *ρ*, anthelmintic treatment rate σ, and faeces removal rate φ represent the proportion of raccoons, parasites and eggs removed from the system in one day to the total number of raccoons, parasites or eggs present on that day. Rates can vary between 0 and 1, with 0 representing the removal of 0 hosts/parasites/eggs, and 1 represents the removal of the whole raccoon/parasite/egg population (100%). In order to comply with practical needs, when simulating raccoon depopulation in the native scenario we did not consider the possibility of host extinction, whereas in case of an introduced population we analysed both possibilities: the reduction of raccoon population without extinction, and the extinction of raccoons. Performing an analysis of the equilibria of the system (S1 Text) based on the actual parameters, the boundary of *ρ* discriminating between these two situations resulted 3.97x10^-3^, meaning that by removing more than 0.397% hosts/day, the host population goes towards extinction.

The full list of parameter values is given in Table 1, the time unit used is one day.

**Table 1:** Parameters included in the models.

Parameter	Interpretation	Value	Source
H	Total number of host population	-	-
P	Total number of parasite population	-	-
E	Total number of free-living infective stage population (eggs)	-	-
K	Host population carrying capacity	1000	-
b	Instantaneous birth rate of host (day^-1^)	0.0052	3.8 young/female/year [35]
d	Instantaneous death rate of host due to all causes except parasites (day^-1^)	0.00123	2.3 years lifespan [36]
μ_1_	Instantaneous death rate of adult parasite (day^-1^)	0.001369	1–2 years lifespan [41,42]
μ_2_	Instantaneous death rate of adult parasite due to density dependent effects (day^-1^)	0.0009	-
h	Instantaneous rate of production of infective parasite eggs (worm^-1^day^-1^)	179 000	179000 eggs/worm/day [21,44]
β	Instantaneous rate of ingestion of free-living infective eggs (host^-1^day^-1^)	4.25e-12	-
k	Aggregation parameter of the negative binomial distribution	0.393	[44]
δ	Instantaneous death rate of eggs (day^-1^)	0.015	-
ρ	Host removal rate (day^-1^)	-	-
σ	Anthelmintic efficacy (day^-1^)	-	-
φ	Eggs removal rate (day^-1^)	-	-

### Sensitivity analysis on *ρ*, *σ* and *φ*

To evaluate the effect of intervention strategies, we performed a global sensitivity analysis to determine the effect of parameters *ρ*, *σ* and *φ* on the number of environmental eggs in the native population scenario. Since we did not investigate possible combinations of intervention strategies, the sensitivity analysis was carried out by moving one parameter at a time. Parameters *σ* and *φ* were left free to vary between their minimum and their maximum (i.e. simulating the elimination of 0–100% population/day) following a uniform distribution. The parameter *ρ* was left free to vary between 0 and 0.00397, in order to avoid the extinction of hosts and the consequent shift from the system of three populations (Eq 2) to the system with only the egg population. The final mean egg number (averaged over the simulation interval) was used to evaluate the effects of the changes in parameter values. The simulation interval for the sensitivity analysis was considered as 50 years, and simulations were performed 500 times for each intervention strategy. Global sensitivity analysis was carried out using the function “modCRL” of the package FME on R software [32].

## Results

### Dynamics of the system

**Fig 2:**
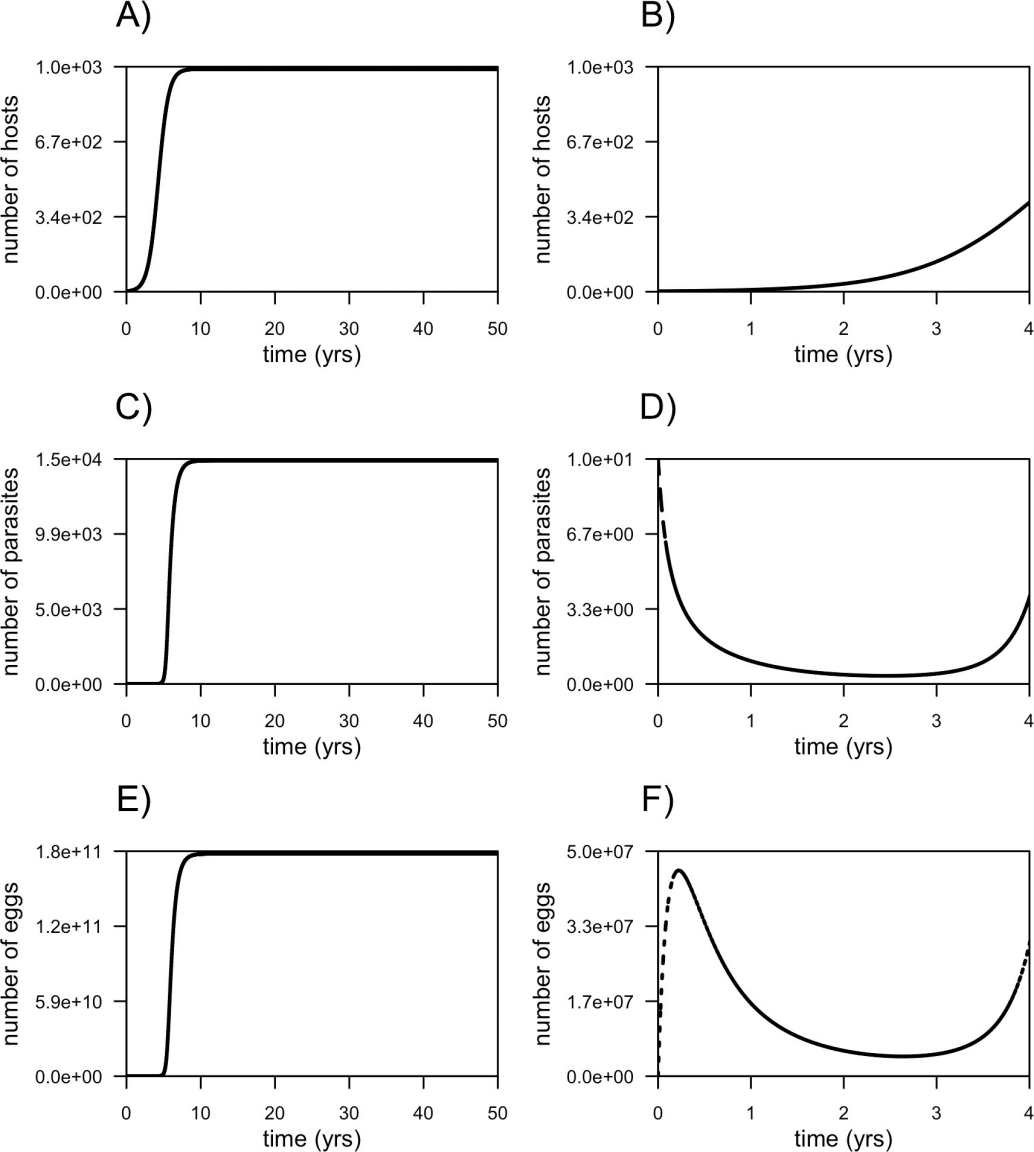
Raccoons-*Baylisascaris procyonis* dynamics: the base model. Temporal dynamics of raccoon population and B. procyonis until the achievement of the steady state of the system (A, C and E); and until the host population reaches 500 individuals (B, D and F).

The base model, in the absence of human intervention and after the introduction of two raccoon individuals with a parasite intensity of 5 *B*. *procyonis* each, predicts a globally sigmoid growth curve for all the three populations (Fig 2). However, the dynamics of parasite and egg populations in the first four years show a slight decrease in their size followed by a sigmoid growth (Fig 2D and 2F).

Simulations indicate that the base system reaches the equilibrium around 20 years, when the raccoon population counts 1000 individuals, the parasite population 14,772 parasites and the egg population 1.7x10^11^ eggs (Fig 2A, 2C and 2E). The 50% of the carrying capacity for the host population (500 raccoons) is reached in about 4 years from the beginning of the simulations, with a total number of 11 parasites and 7.97x10^7^ eggs (Fig 2B, 2D and 2F).

### Sensitivity analysis

**Fig 3:**
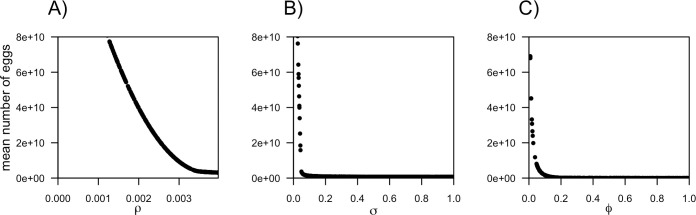
Sensitivity analysis: Sensitivity analysis of parameters ρ, σ and φ on the mean egg number.

The sensitivity analysis showed that all the three parameters can affect the number of eggs. The parameter which variation has the greatest impact on the egg number is host removal (ρ), followed by parasite removal (σ) and lastly egg removal (φ) (Fig 3).

### Intervention strategies in the “native population scenario”

#### Raccoon depopulation

**Fig 4:**
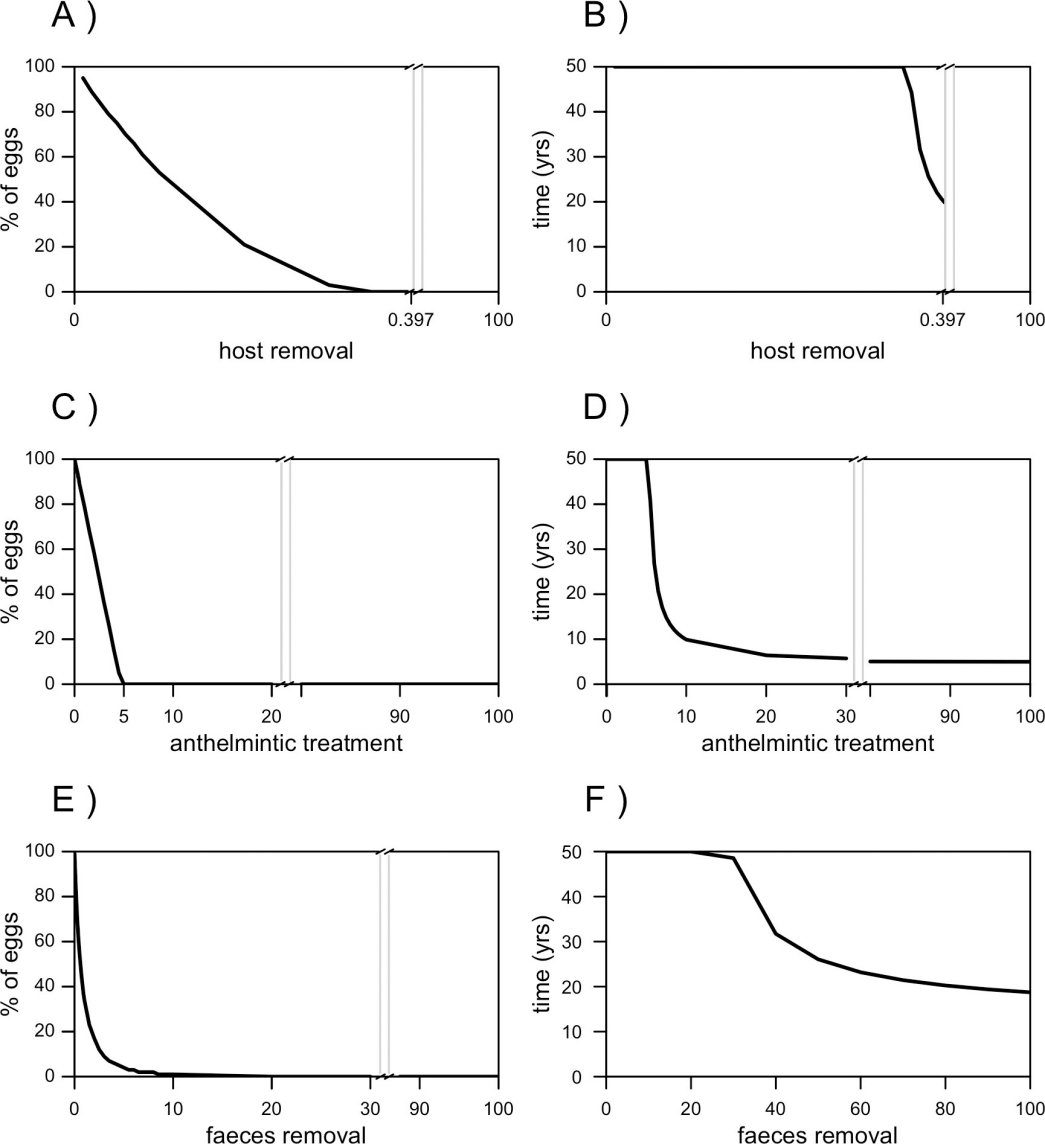
Performance of intervention strategies against *Baylisascaris procyonis*: The native population scenario. Efficacy (expressed as percentage of persisting eggs) and efficiency (expressed as time needed to reach the steady state of the system) of host removal (A and B), anthelminthic treatment (C and D) and faeces removal (E and F).

Without bringing the host population to extinction (i.e. host removal between 0 and 0.397% hosts/day), a removal of less than 0.36% raccoons/day leads to a progressive reduction, but not to the elimination, of the egg population (Fig 4A and 4B). When removing more than 0.36% raccoons/day, the elimination of the egg population is achieved and the higher is the coverage, the faster is their elimination (Fig 4B). Coverages between 0.39 and 0.397% raccoons/day lead to the elimination of the egg population in about 20–22 years (Fig 4B).

#### Anthelmintic treatment

Any treatment coverage that removes more than 5.5% parasites/day leads to the egg population elimination in less than 50 years (Fig 4C). Coverages that remove more than 7.5% parasites/day lead the egg population to zero in less than 20 years, and to reach the elimination of eggs in less than ten years it is necessary to use coverages that remove more than 10% parasites/day. The elimination of eggs can be achieved between 5 and 6 years with coverages higher than 30% parasites/day (Fig 4D).

#### Environmental faeces removal

Treatment coverages that remove more than 50% eggs/day lead to the elimination of the egg population in less than 50 years, but the elimination of eggs through faeces removal can never be reached in less than 19 years (Fig 4F).

### Intervention strategies in the “introduced population scenario”

#### Raccoon depopulation

**Fig 5:**
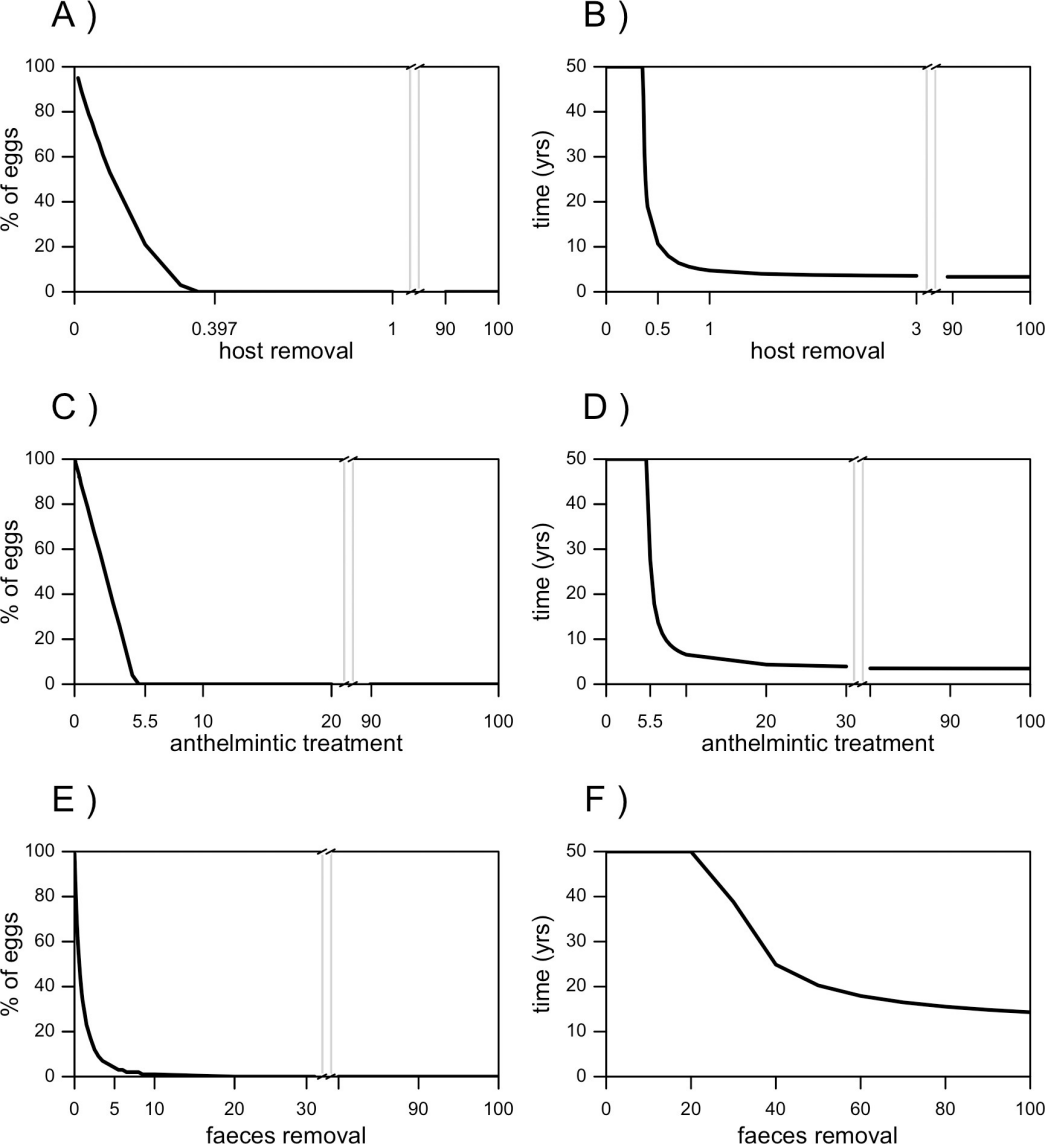
Performance of intervention strategies against *Baylisascaris procyonis*: The introduced population scenario. Efficacy (expressed as percentage of persisting eggs) and efficiency (expressed as time needed to reach the steady state of the system) of host removal (A and B), anthelminthic treatment (C and D) and faeces removal (E and F).

Without bringing the host population to extinction (i.e. by removing less than 0.397% hosts/day), we need to remove more than 0.36% hosts/day to achieve the elimination of the egg population in less than 50 years (Fig 5A), and the time needed to achieve it, is included between 19 years and 43 years (Fig 5B). Within this range of coverages (from 0 to 0.397% hosts/day), the efficiency of the treatment increases linearly, with a progressive reduction in the time needed to eliminate eggs up to a minimum of 19 years. Assuming that the complete extinction of raccoons is instead an allowed outcome, the more hosts/day we remove, the faster we can reach the equilibrium, with a minimum of 3 years needed for egg elimination when removing 100% hosts in one day. Removing between 0.397 and 0.5% hosts/day we need more than 10 years to eliminate the egg population, whereas when removing more than 0.5% hosts/day less than 10 years are required. (Fig 5B).

#### Anthelmintic treatment

By using an anthelmintic drug treatment, we can achieve the elimination of the egg population in less than 50 years only when applying coverages that remove more than 5.5% parasites/day (Fig 5C and 5D). At such rates, the higher are the drug treatment rates, the faster is the elimination of eggs (Fig 5D). To eliminate eggs in less than 20 years, it is necessary to use a coverage that removes more than 6% parasites/day, and to do it in less than 10 years, a coverage that removes more than 7.5% parasites/day. Rates that remove more than 30% parasites/day take 3–4 years to reach the elimination of eggs (Fig 5D).

#### Environmental faeces removal

Using faeces removal as a control strategy, the elimination of the environmental egg population is achieved in less than 50 years with removal coverages that remove more than 30% eggs/day, and in less than 20 years with coverages that remove more than 50% eggs/day (Fig 5F).

### Comparison of intervention strategies

The sensitivity analysis shows that all the examined intervention strategies can eliminate the *B*. *procyonis* egg population (Fig 3). Additionally, the comparison of the efficacy of the three intervention strategies (Table 2) shows that all the three techniques can eliminate the egg population within a 50-years time frame. However, their efficiency varies largely depending on the simulated treatment coverage: raccoon depopulation requires the lowest coverage both to reach egg elimination and to allow it in the shortest time, while faeces removal requires the highest treatment coverage. Anthelmintic treatment has an intermediate efficiency.

**Table 2:** Comparison of years needed to eliminate *Baylisascaris procyonis* egg population by applying different intervention strategies (host removal, anthelminthic treatment and faeces removal) with different treatment coverages (i.e. percentage of hosts/parasites/eggs removed per day), on both native and introduced raccoon (*Procyon lotor*) host populations.

Treatment coverage (%)	Native population			Introduced population		
	Time (yrs) to reach equilibrium through:			Time (yrs) to reach equilibrium through:		
	host removal^a^	anthelmintic treatment	faeces removal	host removal	anthelmintic treatment	faeces removal
**0.01**	no egg elimination^c^	no egg elimination^c^	no egg elimination^c^	no egg elimination^c^	no egg elimination^c^	no egg elimination^c^
**0.05**	no egg elimination^c^	no egg elimination^c^	no egg elimination^c^	no egg elimination^c^	no egg elimination^c^	no egg elimination^c^
**0.1**	no egg elimination^c^	no egg elimination^c^	no egg elimination^c^	no egg elimination^c^	no egg elimination^c^	no egg elimination^c^
**0.2**	no egg elimination^c^	no egg elimination^c^	no egg elimination^c^	no egg elimination^c^	no egg elimination^c^	no egg elimination^c^
**0.3**	no egg elimination^c^	no egg elimination^c^	no egg elimination^c^	no egg elimination^c^	no egg elimination^c^	no egg elimination^c^
**0.35**	no egg elimination^c^	no egg elimination^c^	no egg elimination^c^	no egg elimination^c^	no egg elimination^c^	no egg elimination^c^
**0.36**	44	no egg elimination^c^	no egg elimination^c^	43^b^	no egg elimination^c^	no egg elimination^c^
**0.37**	30	no egg elimination^c^	no egg elimination^c^	31^b^	no egg elimination^c^	no egg elimination^c^
**0.39**	22	no egg elimination^c^	no egg elimination^c^	21 ^b^	no egg elimination^c^	no egg elimination^c^
**0.396**	20	no egg elimination^c^	no egg elimination^c^	20 ^b^	no egg elimination^c^	no egg elimination^c^
**0.397**	20	no egg elimination^c^	no egg elimination^c^	19 ^b^	no egg elimination^c^	no egg elimination^c^
**0.4**	-	no egg elimination^c^	no egg elimination^c^	19	no egg elimination^c^	no egg elimination^c^
**0.5**	-	no egg elimination^c^	no egg elimination^c^	11	no egg elimination^c^	no egg elimination^c^
**0.8**	-	no egg elimination^c^	no egg elimination^c^	6	no egg elimination^c^	no egg elimination^c^
**1**	-	no egg elimination^c^	no egg elimination^c^	5	no egg elimination^c^	no egg elimination^c^
**3**	-	no egg elimination^c^	no egg elimination^c^	3	no egg elimination^c^	no egg elimination^c^
**5**	-	no egg elimination^c^	no egg elimination^c^	3	no egg elimination^c^	no egg elimination^c^
**5.5**	-	41	no egg elimination^c^	3	28	no egg elimination^c^
**7.5**	-	15	no egg elimination^c^	3	10	no egg elimination^c^
**10**	-	10	no egg elimination^c^	3	7	no egg elimination^c^
**30**	-	6	49	3	4	39
**50**	-	5	26	3	4	20
**80**	-	5	20	3	3	16
**100**	-	5	19	3	3	14

^a^ treatment coverages causing hosts’ extinction has not been investigated

^b^ no host extinction

^c^ elimination of the egg population/equilibrium of the system is not reached within 50 years

## Discussion

In the present study, by modelling host-parasite interactions, we analysed the efficacy and the efficiency of alternative intervention strategies to control the environmental persistence of a zoonotic macroparasite infective stage. The analyses showed that both efficacy and efficiency of the intervention primarily depend on the treatment coverage, and only secondary on the chosen treatment and scenario.

The lack of background information when dealing with neglected emerging diseases represents a challenge when assessing control measures, in particular for wildlife originated diseases, since monitoring of wildlife species is often limited [8,11]. The use of mathematical modelling can be helpful to understand the dynamics underlying infectious diseases, providing a tool for an *a priori* evaluation of such dynamics and interactions, and widely contributing to the design of control programs of diverse infections, without the need of demanding empirical studies [18,34,46,47]. For instance, the use of mathematical models in the development of control programs for measles, pertussis or rubella produced useful predictions concerning the level of vaccination coverage required to eradicate them, helping in the determination of the relative merits of different policies for the control of these infections [45,48]. Despite this, mathematical models are still largely underexploited in the planning of public health policies and disease control strategies [20].

Our work aimed at supporting the use of mathematical models as a pre-intervention approach to assess the effectiveness of different control strategies against emerging diseases. By simulating the effect of different intervention strategies on parasite population dynamics, through their direct inclusion into the system, this approach provides results that can serve as a base for the *a priori* evaluation of an intervention strategy program.

While we used raccoons and their zoonotic helminth *B*. *procyonis* as a model system to identify the most effective intervention strategy to reduce parasitic environmental contamination, our simulations can be easily generalized to different macroparasitic diseases, including most diseases caused by ascarids and other soil-transmitted helminths. We chose to use a pre-existing and already validated model to highlight how even the application of simple and existing models can provide useful information about systems. This holds especially true when parameters and biological processes are hard to estimate due to a lack of field data, and any modification of base models can be both challenging to perform and less informative than simpler models. Moreover, the use of a validated model can extend the use of mathematical modelling to a wider spectrum of research areas, not limited to mathematicians and field experts.

With respect to the specific simulations performed on the raccoon-*B*. *procyonis* system, in both the native and introduction scenario all the treatments were potentially effective in reaching the elimination of the egg population within 50 years, but both their efficacy and efficiency varied greatly depending on the applied treatment coverage. The performed sensitivity analysis indicated host removal as the most effective strategy, while faeces removal had the lowest impact on the number of eggs. In agreement with this, the analysis of the intervention strategies via model simulations showed that faeces removal is the less efficient intervention strategy, as both host removal and anthelmintic treatment were faster in eliminating the egg population. However, anthelmintic treatment requires a higher effort than host removal to reach an effective result. Ultimately, host depopulation is thus the treatment that requires the lowest treatment coverage to provide the elimination of eggs in both native and introduction scenarios and, together with anthelmintic treatment, shows also faster results. However, the time needed to reach the elimination of eggs depends on the simulated scenario. This result highlights the importance of taking into account differences between scenarios when choosing the intervention strategy to apply, although choosing an appropriate treatment coverage remains the most important step to achieve the elimination of the egg population. Moreover, the great difference in the efficiency of treatments resulting from even a slight change in coverages, as it happens between 0.37 and 0.39% hosts/day for host removal or between 5.5 and 7.5% parasites/day for anthelmintic treatment, demonstrates the importance of an *a priori* evaluation of the effects of intervention strategies.

Currently, raccoon depopulation and anthelmintic treatment are indeed the most frequently applied intervention strategies to reduce *B*. *procyonis* environmental contamination, and many authors suggested them as the most effective techniques [21,26,30,31]. However, a formal framework to objectively assess their efficacy and efficiency was lacking. With our model, we provide a quantitative analysis of both the efficacy and efficiency of these strategies, providing indications about the effort needed to reach the desired result without wasting time and economic resources.

However, it must be underlined that the choice of the most appropriate intervention strategy cannot overlook the need of an accurate analysis of its logistical feasibility under field conditions. A mathematical model can provide information to identify the most efficient method, but its application also needs the participation of field scientists and technicians, to evaluate the logistical feasibility and applicability of the intervention strategies through a cost-benefit analysis.

However, for a more complete outline of an intervention strategy, combinations of different treatments, or of discontinuous treatments (such as a monthly administration of anthelmintic baits) should be simulated. A continuous daily treatment, as we simulated, allowed for a more explicit comparison between intervention strategies, but when assessing an intervention strategy, it is advisable to include a realistic time frame between two consecutive treatments. Finally, in addition to the insights provided by the model on *B*. *procyonis* control, the base model simulating raccoons-*B*.*procyonis* dynamics without any human intervention provides interesting information about the system as well. For instance, the simulation of the base model in the introduction scenario shows that the adult parasite population growth is markedly slower than the growth of both host and egg populations. Indeed, based on our estimates, while both host and egg population sizes increase very fast soon after simulating the introduction of raccoons, the number of adult parasites initially decreases. This relevant difference in the dynamics of the three populations highlights the need of a deeper analysis of the dynamics of the system at the early stages of introduction. When dealing with recently established raccoon populations, an early detection of *B*. *procyonis* is indeed fundamental to limit environmental contamination and reduce the infection risk for humans. However, the low parasite abundance during the first stages of raccoon invasion suggested by our simulations could hinder *B*. *procyonis* detection and must therefore be taken into account when implementing surveillance plans. This unexpected dynamic of the parasite population could depend on the initial low number of hosts eliminating eggs, which will in turn determine a small egg population and overall a low parasite transmission rate. The inclusion of a stochastic model to predict more accurately the early stages of raccoon introduction, and the inclusion of specific field data can represent an implementation of the study, allowing for a more precise estimate of biological and epidemiological parameters, resulting in a more detailed and realistic simulation of population dynamics and in the inclusion of diverse mortality or transmission rates for adult parasites and eggs. Finally, we focussed our study on the effects of the intervention strategies on the system, but a wider sensitivity analysis including rates representing parameters other than ρ, σ and φ could be performed to further investigate which biological processes affect the base system the most. In conclusion, our simulations suggest host depopulation as the most efficient strategy to control environmental contamination by *B*. *procyonis* eggs, but they also highlight that, no matter the chosen technique, the treatment coverage is the most important parameter determining the effectiveness of control strategies. This work highlights the potential benefits of applying mathematical modelling in epidemiology and public health management, showing their efficiency as a tool to analyse disease dynamics and implement time- and cost-effective intervention strategies, even when a complete knowledge about the system is lacking and an empirical approach is unpractical.

## Supporting information

S1 Text System for analytical computation of equilibria. (DOCX)
