# Supplementary material for: How to choose the best control strategy? Mathematical models as a tool for pre-intervention evaluation on a macroparasitic disease
Source: PLoS Negl Trop Dis. 2020 Oct 22;14(10):e0008789. doi: 10.1371/journal.pntd.0008789 (PMC7608949; doi:10.1371/journal.pntd.0008789)
Supplement: S1 Text — (DOCX) [file pntd.0008789.s001.docx]

**S1 Text. System for analytical computation of equilibria**

$$\left\{ \begin{aligned} \hat{H}=K\frac{(b-d-\rho)}{(b-d)} \\ \hat{P}=(\frac{\hat{H}\beta h}{\hat{H}\beta+\varphi\delta}-\left( \sigma+\mu_{1}+d+\rho+\mu_{2} \right))(\frac{\hat{H}k}{\mu_{2}(k+1)}) \\ \hat{E}=\frac{h\hat{P}}{\hat{H}\beta+\varphi+\delta} \end{aligned} \right. (1)$$

Where $\hat{H}$, $\hat{P}$ and $\hat{E}$ respectively represent the number of hosts, parasites and eggs at equilibrium.
